# Three mitochondrial genomes of freshwater fishes in the genus *Squalidus* (Cypriniformes: Gobionidae)

**DOI:** 10.1080/23802359.2020.1835583

**Published:** 2020-11-20

**Authors:** Jie Chai, Cuizhang Fu

**Affiliations:** Ministry of Education Key Laboratory for Biodiversity Science and Ecological Engineering, Coastal Ecosystems Research Station of the Yangtze River Estuary, Institute of Biodiversity Science and Institute of Eco-Chongming, School of Life Sciences, Fudan University, Shanghai, PR China

**Keywords:** Cypriniformes, East Asia, Gobionidae, *Squalidus*

## Abstract

Mitochondrial genomes of *Squalidus mantschuricus*, *S. chankaensis*, and *S. longifilis* have been determined using Sanger sequencing (GenBank Accession No. MT767745–MT767747). The three mitochondrial genomes consist of 13 protein-coding genes, two *rRNA* genes, 22 *tRNA* genes, and one control region with the length of 16,605, 16,611, and 16,607 bp. Phylogenetic analysis of the three species showed that *S. mantschuricus* is nested within a fully supported terminal clade with *S. argentatus*, and *S. chankaensis* is a sister group of *S. mantschuricus*, *S. argentatus*, and *S. wolterstorffi*. *Squalidus longifilis* is positioned in a clade with *S. multimaculatus* and *S. gracilis*.

An updated classification system of the order Cypriniformes proposed by Tan and Armbruster (2018) places the genus *Squalidus* into the family Gobionidae. *Squalidus* fishes are comprised about 14 species (Fricke et al. 2020), and they mainly live in streams and rivers of East Asia (Yue 1998). *Squalidus longifilis* is treated as valid species in a recent study (Chai 2020). In this study, three mitochondrial genomes were obtained using Sanger sequencing: *S. mantschuricus*, *S. chankaensis*, and *S. longifilis*. These data are useful for species delimitation and phylogenetic reconstruction.

*Squalidus mantschuricus* (voucher FDZM-SqMDH20110719-01) was sampled from Dunhua City, Jilin province, China (43.69°N, 128.60°E), *S. chankaensis* (FDZM-SqCJal20100719-01) from Jalaid Banner, Inner Mongolia Autonomous Region, China (46.78°N, 122.68°E), and *S. longifilis* (FDZM-SqLFengC20170820-01) from Fengcheng County, Liaoning province, China (40.46°N, 124.11°E). All specimens were deposited in the Zoological Museum of Fudan University (FDZM), China. Total genomic DNA was extracted from muscle tissue using a high-salt method (Miller et al. 1988). Thirteen pairs of primers were designed to amplify contiguous, overlapping segments using the polymerase chain reaction (PCR). The primers were as follows: Gob12sF 5′-AAGGCATGGTCCYGACCTTA-3′ and Gob16sR 5′-TTCGGTAGGTCTRTCACTTC-3′ (Primer1); Gob16sF 5′-ACCTTGTACCTTTTGCATC-3′, and GobLeuR 5′-GGGAA

GAGGAYTTGAACC-3′ (Primer2); GobND1F 5′-GCAGCCGCTATTAAGGGTT-3′ and GobND1R 5′-GGRTTCATTGATGGAGGA-3′ (Primer3); GobIleF 5′-GCCCAAG GACCACTTTGATAG-3′ and GobCOIR 5′-CCAAATACRAGATARAGGT-3′ (Primer4); GobAsnF 5′-AGCGAGCATCCATCTACTT-3′, and GobSerR 5′-GGTYATG

TGACTGGCTTGA-3′ (Primer5); GobCOIF 5′-TGAGAAGCCTTYGCCGCYAAACG′-3′ and GobATP6R 5′-AGGAATACYATYAGGGAGGC-3′ (Primer6); GobATP6F 5′-CCTTGAGAYTGACCATGAT-3′ and GobArgR 5′-CTGAGYCGAAATCAGAGG-3′ (Primer7); GobCOIIIF 5′-TGATGAGGCTCATATCTTTCTA-3′ and GobND4R 5′-TCTGTGGCRCCRAATGCTAT-3′ (Primer8); GobND4F 5′-TAGCATTTCAYCGC ACMC-3′ and GobLeuR 5′-TGGAYTTGCACCAAGAGT-3′ (Primer9); GobSerF 5′-ACTYACCRAGGAAGGACA-3′ and GobND5R 5′-TCCYCAGGCAAGYCGTTT-3′ (Primer10); GobND5F 5′-ATTGARGCCCTAAACACCTC-3′, and CGobCytbR 5′-AA GTGGAAKGCGAARAATCG-3′ (Primer11); GobND6F 5′-AAAATAGGTCATAA

TTCTTGCTCGG-3′ and GobProR 5′-GTTTAATTTAGAATTCTGGCTTTGG-3′ (Primer12); GobDloopF 5′-AAAGCATCGGTCTTGTAATC-3′, and GobDloopR 5′-CTTGGCTAGGCGTCTTGG-3′ (Primer13). Thermocycling parameters were comprised 35 thermal cycles, denaturation at 94 °C for 50 s, annealing at 52.6–55.6 °C for 60 s, and extension at 72 °C for 70 s on a 2720 Thermal Cycler (Applied Biosystems, Foster City, CA). The PCR products were purified and directly sequenced using the PCR primers in an ABI 3730 DNA sequencer (Applied Biosystems, Foster City, CA). The assembly and annotation of mitochondrial genomes followed the methods published for *Pseudorasbora elongata* by Chen et al. (2015). The maximum likelihood (ML) analysis was inferred using five partitions (each codon of all protein-coding genes, *12S + 16S rRNA* genes, and all *tRNA* genes) and IQ-TREE version 1.6.2 (Nguyen et al. 2015) with 1000 ultrafast bootstraps (UFBoot) (Hoang et al. 2018). The best substitution model was estimated with ModelFinder (Kalyaanamoorthy et al. 2017) following the BIC criterion.

The lengths of new mitochondrial genomes are 16,605, 16,611, and 16,607 bp for *S. mantschuricus*, *S. chankaensis*, and *S. longifilis*, and contain the A + T base compositions of 56.0, 55.5, and 54.0%, respectively. Gene compositions, codon uses and gene arrangements are similar to previously published gobionid mitochondrial genomes (Chen and Fu 2019; Fu and Fu 2020; Yi and Fu 2020). For 13 protein-coding genes, start codons include ATG or GTG, and stop codons consist of TAA, TAG, TA–, or T—. Results of the inferred ML tree (Figure 1) show that the genus *Squalidus* is a monophyletic taxon. *Squalidus mantschuricus* is nested in a clade with *S. argentatus* with high bootstrap support. *Squalidus chankaensis* is a sister species to *S. mantschuricus*, *S. argentatus*, and *S. wolterstorffi*, and is fully supported. *Squalidus longifilis* and *S. multimaculatus* are positioned in a well-supported clade with *S. gracilis*.

**Figure 1.**
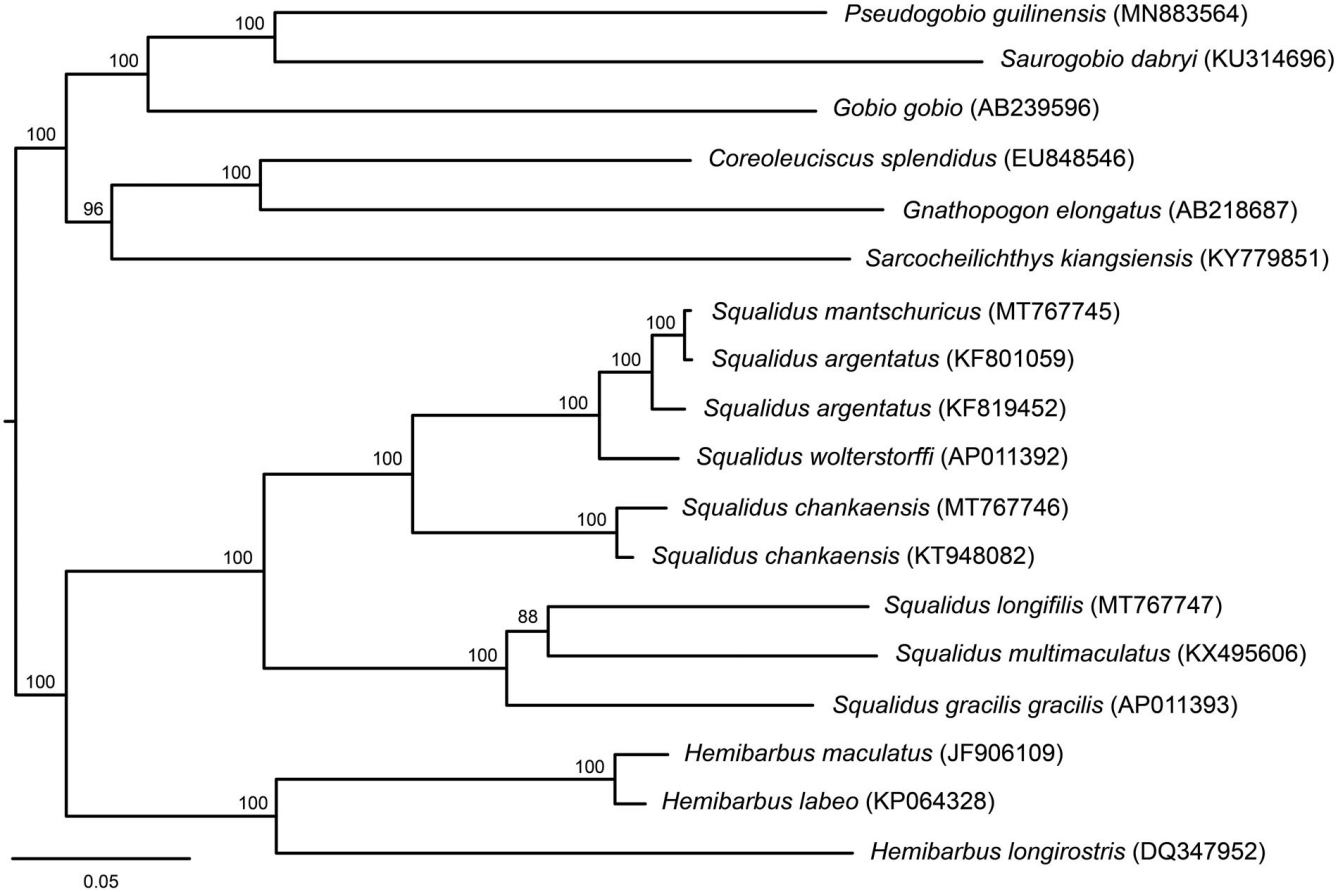
An IQ-TREE tree of *Squalidus* fishes and their close relatives using a maximum likelihood analysis with GenBank numbers in the parentheses and bootstrap confidences above branches.

## Data Availability

Three new mitochondrial genomes with accession numbers have been released in the GenBank: MT767745 (https://www.ncbi.nlm.nih.gov/nuccore/ MT767745), MT767746 (https://www.ncbi.nlm.nih.gov/nuccore/ MT767746), and MT767747 (https://www.ncbi.nlm.nih.gov/nuccore/ MT767747).
